# Progress of the Medical Sciences

**Published:** 1914-12

**Authors:** 


### Progress of the Medical Sciences.

### MEDICINE.

**Diabetes mellitus.** The well-known fact that in severe cases of diabetes the urinary sugar is only reducible to a certain point by restricting the carbohydrate intake, has been shown by Wolf and Guttmann [1] to bear an interesting relationship to the sugar content of the blood. In uncomplicated and mild cases the blood-sugar falls to normal on appropriate diet, but as the cases increase in severity the point to which the blood-sugar can be reduced mounts higher and higher, and the minimum content for each individual patient is uninfluenced by further decrease of the sugar intake. When the urinary sugar had been reduced to zero by means of " vegetable days," in which both protein and carbohydrate were taken in very small quantities, the hyperglycæmia remained at its accustomed minimal figure, and slight increases on this amount were observed on " oatmeal " days. The blood-sugar also rose in cases of coma. Complications such as phthisis, arteriosclerosis and cirrhosis of the liver did not influence the behaviour of sugar content of the blood, but in chronic granular kidney there was increased hyperglycæmia even when the urine was permanently free from sugar, and in other cases of nephritis variations on the generally-found blood content were observed. That valuable information may be obtained by estimations of the sugar content of the blood is thus clear, but unfortunately the methods for doing so are difficult and not always exact.

Klercker,[2] also making estimations of the sugar content of the blood, found that the exhibition of the opium alkaloids had no influence on experimental hyperglycæmia induced by injections of adrenin, or by diabetic puncture. On the other hand, when alkaloids were injected in amounts which failed to influence the normal sugar content, feeding with such amounts of carbohydrate as would ordinarily exceed the animal's level of toleration failed to produce hyperglycæmia.

It has been suggested[3] that this effect on " alimentary glycosuria " may be due to a retarding influence on the rate at which the stomach empties itself, which has been shown by Magnus to be one of the results of the administration of morphia. The carbohydrates may thereby be prevented from reaching the small intestine in large quantities at a time, and thus absorption being likewise diminished, no hyperglycæmia results.

Galambos and Tansz[4] attribute hyperglycæmia to a diminution in the internal secretion of the pancreas or to some change in its activity, which interferes with the normal katabolism of proteins. Analogous to the glycosuria is a hyperaminosuria, or increased secretion of amino-compounds in the urine, which they have shown to occur in pancreatic insufficiency. A similar condition is also found in some liver diseases and in certain infections in which glycosuria may also be observed.

**Nervous glycosuria.** It has long been known to clinicians that emotional disturbances, especially fear and anger, can produce or increase glycosuria ; recently experimental evidence has been produced showing that glycosuria occurs in cats when they are confined in cages and frightened by dogs.[5] The reaction depends on the actual fear or rage experienced by the cats ; an interesting fact brought out by these experiments was that no glycosuria occurred if the suprarenals had previously been removed, which corroborates the view that painful emotions are accompanied by an increased discharge of the internal secretion of these glands into the blood.

**Renal glycosuria** has been critically reviewed by de Langen, [6] who also gives a detailed account of one case. He remarks that the absence of carefully-observed cases is probably due to the fact that the glycosuria is usually slight, and patients are thus seldom admitted to hospitals. In his own case, a neurasthenic young man of 20 years of age, with a neuropathic family history, the amount of sugar was, within wide limits, entirely uninfluenced by diet. The glycæmia was uniformly low, and also independent of the sugar intake. In the period of more than twelve months during which he was under observation, the general condition improved considerably, but the glycosuria and sugar tolerance remained practically uninfluenced.

**Diabetic coma.** The theories as to the causation of diabetic coma have recently been summarised as follows :[7] Klemperer and von Noorden thought that both coma and β-oxybutyric acid were the product of an unknown toxic agent, as they are associated in various other conditions, for example in fever and starvation ; in anæmic states and sometimes in dyspepsia acetone, diacetic acid and occasionally β-oxybutyric acid are excreted in increased amounts, and drowsiness may be present. On the other hand, the last-named product may be excreted apart from a comatose condition in uræmia and hysteria. Stadelmann and Naunyn do not consider that there is any specific action by the acetone bodies, but believe that the coma is merely due to the elimination of sodium bases. While admitting that the acetone bodies act specially on the vasomotor, respiratory and higher cortical centres, Nasel does not believe that a true acid intoxication occurs, such as is found, for instance, in narcosis where the hydrogen ions are really in excess in the blood. Patients with a pronounced degree of the so-called " acidosis " have the same concentration of hydroxyl ions in their blood as normal individuals, and some gouty people have a less concentration than diabetics. Hence the occasional deficiency in the latter must be merely a secondary effect of disturbed metabolism.

Blum [8] has investigated the relationship between coma and blood-pressure, and finds that the latter varies. At the beginning it is frequently normal, and then usually falls, but may occasionally rise in the course of the attack. Hypotonia of the medullary centres is usually a late symptom ; it does not depend on the height of the blood-pressure, and is apparently due to changes in the content of the tissues in water and salts.

Both clinically and experimentally it can be shown that the hypotonia is not a specific symptom of diabetic coma or the action of the acetone bodies.

**Diabetes insipidus.** Römer[9] has reported a case of this disease in a child aged 9 years, in which Aubing's colloid extract of the pituitary gland of the ox was administered and followed within a few hours by decreased secretion of urine, and partial disappearance of the comatose state which had previously existed. The child, however, died, and was found to have a tumour which compressed the posterior lobe and the infundibulum. He also performed certain experiments on rabbits, which showed that this extract caused diminished flow of urine. Pituitrin and Pituglandol, somewhat similar preparations, had the same effect though in less marked degree. He therefore agrees with von den Velden and Farmi that diabetes insipidus is in some cases due to deficient pituitary secretion.

Camus and Roussy,[10] on the other hand, as the result of operative procedures on animals (dogs), have shown that the destruction of an area at the base of the brain close to the infundibulum will produce polyuria when the latter structure itself is intact. Removal of the hypophysis alone did not produce polyuria.

J. M. Fortescue-Brickdale.

REFERENCES.

1 *Ztschr. f. Klin. Med.*, 1914, lxxix. 394.

2 *Biochem. Ztschr.*, 1914, lxii. 11.

3 *J. Am. M. Ass.*, 1914, lxiii. 786.

4 *Ztschr. f. Klin. Med.*, 1914, lxxx.

5 Cannon Shohl and Wright, *Am. J. Physiol.*, 1911, xxix. 283.

6 Abstract in *Schmidt's Jahrbuch*, 1914, cccxix. 570.

7 *Med. Rec.*, 1914, lxxxv. 204.

8 *Berl. Klin. Wchnschr.*, 1913, l. 2135.

9 *Deutsche. Med. Wchnschr.*, 1914, xl. 108.

10 *Compt. Rend. Soc. de Biol.*, 1914; abstract in *Lancet*, 1914, ii. 513.

### SURGERY.

**Fracture of the Neck of the Femur.**—The unsatisfactory nature of the results of intra-capsular fracture of the femur, especially in old people, is well known. Royal Whitman[1] maintains that repair is possible in every variety of this fracture, provided the fragments are in contact. The object of treatment, therefore, is to provide opportunity for repair, and he claims that the abduction treatment is the only method that enforces

[1] *Ann. Surg.*, 1914, lx. 262, 485, *et seq.*

this principle. Whitman's method, which is not sufficiently well known, is carried out as follows in cases of complete fracture : The patient, generally under an anæsthetic, is lifted to a pelvic support, preferably furnished with a perinæal bar for counter-pressure. The extended limbs are supported by assistants. The assistant holding the uninjured limb abducts it to the normal limit in order that it may serve as a guide and to fix the pelvis. The injured limb is first flexed and rotated sufficiently to disengage any soft parts that may be interposed between the fragments. It is then completely extended, and the assistant by pulling on the limb, overcomes the shortening as demonstrated by measurement, and by the relation of the tip of the great trochanter to Nélaton's line, and at the same time corrects the outward rotation. Still maintaining steady traction, the limb is then abducted by the assistant to correspond with its fellow, the operator meanwhile supporting the joint and lifting the thigh upwards. The pelvis should now be level, and the extended limbs in exact correspondence. A plaster of Paris spica is then applied from the axilla to the toes on the affected side, careful adjustment being made about the pelvis and trochanter. It should completely cover the buttock, and be heavily reinforced beneath the joint and thigh so that it may be effective in holding the limb in its proper place. The complete reduction of the shortening is essential to the success of the method. The limb is lifted because it is often below its proper relation to the trunk. The reason for complete abduction is that in this position the upper end of the lower fragment is turned to meet the lower end of the upper fragment, and as the capsule becomes tense, it directs them towards each other, and finally forces them into contact. The tense capsule acts as an antero-posterior splint, and upward displacement is prevented by the engagement of the outer fragment with the rim of the acetabulum if the fracture is near the head, or by direct contact of the trochanter with the side of the pelvis it it be at the base.

In incomplete or impacted fractures the deformity can be corrected in nearly all cases in the manner described, aided by gentle downward pressure on the trochanter. There is a type of incomplete fracture, however, in which the neck is depressed by the opening of a wedge-shaped interval in its upper border, and in which the deformity is corrected by traction and abduction.

No direct pressure should be permitted for many months, nor until voluntary control has been regained, and until passive movements are relatively free and painless. The most usual misconception of the abduction treatment is that it requires force ; but, practically speaking, no force is required in the cases now under consideration.

Whitman asserts that, as compared with other methods, the abduction treatment is not only efficient, but it has a far wider range of application. The plaster spica being an independent splint, permits any desired change of posture ; and the head of the bed may be elevated in order to lessen the danger of internal congestion, and to increase the blood supply of the injured bone. Pain on movement, bedsores and hypostatic congestion are, therefore, practically eliminated as complications.

The most important and reasonable explanation of failure of union by ordinary treatment is low vitality resulting in an incapacity for repair ; and this is also the most forcible argument for accurate and secure adjustment of the fractured surfaces in order to assure the possibility of union.

In discussing Whitman's method Hotchkiss [1] stated that since adopting the abduction method of treatment his results had been distinctly better. By this method, function is restored and union secured, and in many instances the results were unexpectedly gratifying. Hitzrot [2] has found this method extremely satisfactory in those cases involving the narrow part of the neck, but not involving the trochanter. In some of the latter cases he considers that the results of forced abduction were not always satisfactory. Dr. Dawbarn [3] thinks that in recent cases of fracture of the neck of the femur, while exudate of callus is still possible, the method of Whitman is ideal ; but in late cases, in which the period of exudation has long gone by, he prefers to expose the joint, bring the fragments into apposition, and hold them together by driving an aluminium spike through the neck and into the head of the femur. To excite an exudation of callus, he makes an injection of the glycerite of tannin, which he has found the most efficacious of all plans.

James Swain.

### OPHTHALMOLOGY.

**West's operation for complete lachrymal obstruction (Hall's modification).** Graham and Paton [4] showed two cases illustrating this operation, which consists of the removal of a small circular piece of mucous membrane from the outer wall of the nasal fossa opposite the anterior end of the middle turbinal. Next a rectangular flap of the mucous membrane including this hole is dissected up and turned backwards and upwards so as to lay bare the bony wall opposite the lachrymal sac. The bony wall of the sac is then chiselled away and a probe is passed into

[1] *Ibid.*, p. 508. [2] *Ibid.*, p. 509. [3] *Ibid.*
[4] *Tr. Ophth. Soc. Lond.*, 1914, xxxiv. 102.

the tear-sac through the lower canaliculus, so as to push the mucous membrane lining its inner wall through the hole in the bone. The bulging membrane is then snipped away, and the flap of nasal mucous membrane is replaced so that the holes in the sac-wall, the bone and the nasal mucous membrane coincide. A strand of strong gut or a piece of thin lead wire is passed from the lower canaliculus through the new passage and out through the nose to help to keep the flap in position and the holes in alignment while healing takes place.

The great advantage of the operation is that the canaliculus drainage system is left intact, and of course there is no disfigurement. With a narrow nasal fossa, as from a deviated septum, the operation presents considerable difficulty. The results are said to be permanent.

**Cysts in the anterior chamber arising from the pars ciliaris retinæ.** Holmes Spicer described a case of this very rare condition at a recent meeting of the Royal Society of Medicine. A white spot appeared at the edge of the pupil of a child aged 3 months. At 16 months there were three finger-shaped masses protruding from behind the iris into the pupillary area. Four years later these had disappeared, and a cyst about 4 mm. diameter was floating freely in the anterior chamber. Some of these cysts disappeared and fresh ones formed. When the child was shown some eight months ago there were seven cysts looking like bubbles in the anterior chamber. Ultimately the eye became glaucomatous and was excised.

Pathological examination showed that these cysts originated from the pars ciliaris retinæ. A membrane grew over the ciliary body and the back of the iris, and from the surface of this membrane the cysts grew. One of the most interesting features of these cysts is that they consisted of embryonic retinal tissue *and contained vitreous,* thus supporting the view that the vitreous is derived from the neural epiblast, and is not of mesoblastic origin.

**Ethyl-hydrocuprein** has recently been recommended for corneal ulcers, more particularly for those due to pneumococcal infection. Schur, of Tübingen, has had very good results.[1] At first he used instillations of a one per cent. solution, as well as direct application of the solution to the ulcer, but later he omitted the drops and applied a one or two per cent. solution to the ulcer by means of cotton wool on a probe. He keeps the cotton wool applied to the ulcer for from one to four minutes, until the cornea round the ulcer shows slight opacity. A two per cent. solution has a caustic effect upon the conjunctiva. Solutions can be sterilised without loss of activity, but act best when freshly prepared.

[1] *Ophthalmoscope,* 1914, xii. 314.

**The efficiency of the eye under different systems of lighting.** Ferree[1] investigated (1) the lighting conditions that give the best visual efficiency; (2) the conditions best for continued work; (3) the most comfortable conditions. Three or four hours' work in daylight causes no appreciable loss in visual efficiency. With direct artificial lighting he finds that there is a great loss, and that even with the same degree of illumination, daylight gives far better acuity of vision than direct artificial light. Proper distribution of light and surface brightness (*i.e.* diffusion of light) are of fundamental importance for sustained efficiency. Momentary judgment is misleading; too bright illumination gives a high degree of acuity, with rapid loss of efficiency and subsequent discomfort. Somewhere about 2.2 foot-candles at the point of work is best, but with proper distribution and diffusion of light the retina readily adapts itself to various degrees of illumination. Speaking of conditions obtaining in America, he says, "For the kind of distribution effects given by the majority of our lighting systems . . . . too much light is being used for the comfort and welfare of the eye." Under similar conditions the white light of the metal filament lamp is better than the yellow light from carbon filaments, both as regards acuity and maintenance of efficiency. He attributes the reputed comfort of an oil lamp to its relatively low degree of illumination and not to the quality of the light.

The question of sustained efficiency of vision is of such immense importance that further investigation ought to be conducted with a view to finding the best conditions of artificial illumination.

**The treatment of the earlier stages of senile cataract.** Colonel Henry Smith, I.M.S., the introducer of the operation for the removal of a cataract in its capsule, thinks that many cases of early cataract are amenable to "treatment."[2] He specially calls attention to the fact that his conclusions are the result of experience in India, where senile cataract is excessively frequent, appears at an earlier age, and matures more rapidly than in Europe.

In uncomplicated cases, with the vision reduced to about $\frac{6}{12}$ or $\frac{6}{9}$ as a result of sand-like opacities and striæ, he gives a sub-conjunctival injection of 25 minims of a 1 in 4,000 to 1 in 6,000 solution of cyanide of mercury. The younger the patient the stronger the solution needed to produce the standard reaction. The operation is done under cocaine, and is usually followed by much pain, so a hypodermic of morphia up to half a grain is given one hour previously. Within a month the patient's vision is restored to $\frac{6}{6}$ or even $\frac{6}{5}$. In the case of railway officials under his care, he gives them a month's leave and an injection of

[1] *Ophthalmology*, 1914, x. 660. [2] *Ophthalmoscope*, 1914, xii. 546.

cyanide of mercury, and they are able to resume work at the end of the month. After more than a year's experience of this treatment he has come to regard the results as permanent.

This procedure has been very little adopted by others at present. Some few are using the method advocated by Dor of Lyons, who for some ten years has been recommending eye-baths of iodide of calcium for twenty to thirty minutes daily.

Smith offers the suggestion that the remote cause of cataract in the Punjab is connected with the dietary. The prevalence of stone in the bladder covers roughly the same area as cataract. He points out that this is a grain-eating and not a rice-eating district.

**The use of salvarsan in ophthalmic practice.** In the *Proceedings of the Royal Society of Medicine* [1] there is a very full account of a discussion opened by Lang. Besides being of use in practically all syphilitic affections of the eye, salvarsan has proved of great use in many cases of iridocyclitis, more particularly if of sympathetic origin. It also appears to have been of some definite use in cases of late infection after cataract extraction. It is of interest also to read of a pack of foxhounds in Madras being injected with "606." By being so treated they were saved from a form of piroplasmosis (conveyed to the hounds by an infected tick), which in previous years had carried off practically every hound in the pack.

C. H. WALKER.

### Reviews of Books.

[1] 1913–14, vii., *Ophth. Sect.*, p. 75.

